# Absence of detectable circadian rhythmicity of neurophysin I, a surrogate marker for oxytocin

**DOI:** 10.1007/s11102-026-01670-0

**Published:** 2026-03-25

**Authors:** Cihan Atila, Rakithan Murugesu, Deborah Rudin, Dino Luethi, Matthias E. Liechti, Mirjam Christ-Crain

**Affiliations:** 1 Department of Endocrinology, Diabetology and Metabolism, University Hospital Basel, Petersgraben 4, 4031 Basel, Switzerland; 2 Department of Clinical Research, University of Basel, University Hospital Basel, Basel, Switzerland; 3 Division of Clinical Pharmacology and Toxicology, University Hospital Basel, Basel, Switzerland; 4 Department of Biomedicine, University of Basel, University Hospital Basel, Basel, Switzerland

**Keywords:** Biomarker, Hypothalamic regulation, Posterior pituitary, Suprachiasmatic nucleus, Vasopressin, Sleep-wake regulation, Melatonin, Social behaviour

## Abstract

**Purpose:**

Oxytocin (OXT) plays key roles in social behaviour, stress regulation, and reproductive physiology. OXT deficiency has been described in pituitary disorders and is associated with increased anxiety and impaired prosocial behaviour. Despite growing clinical interest, the circadian regulation of OXT remains poorly defined, largely due to methodological limitations in measuring endogenous OXT. Neurophysin I (NP-I), a stable carrier protein co-released with OXT, represents a reliable surrogate marker. Characterizing the circadian profile of NP-I may therefore provide important insights into oxytocinergic function with implications for diagnostics and future replacement strategies.

**Methods:**

This study is a secondary analysis of a prospective, observational trial conducted in healthy adults under highly standardized conditions. Thirteen participants underwent hourly blood sampling over 24 h following overnight fasting, controlled fluid intake, standardized meals, and regulated light exposure and physical activity. All assessments were performed on the clinical research ward to minimize external influences on hormone secretion.

**Results:**

Thirteen healthy participants (median age 23 years [IQR, 19–24]; 46% female; BMI 22.8 ± 1.6 kg/m²) underwent 24-hour hormone profiling. NP-I concentrations ranged from 50 to 8,607 pg/mL. The overall median NP-I concentration was 822 pg/mL [339–1,392], with a mean of 1,609 pg/mL (2,084). Individual median 24-hour NP-I concentrations ranged from 143 pg/mL [114–186] to 7,103 pg/mL [6,380–7,714]. Across participants, NP-I levels peaked at approximately 05:30 and reached a nadir at approximately 16:30. The estimated oscillation amplitude was small (35 pg/mL) and not statistically significant (95% CI −110 to 181; *p* > 0.05), indicating the absence of a robust population-level circadian rhythm. Female participants exhibited higher NP-I concentrations than males (median 1,372 pg/mL [468–5,344] vs. 687 pg/mL [297–932]), with markedly elevated levels—approximately four- to five-fold higher—in two women using hormonal contraception.

**Conclusion:**

Under resting, unstimulated conditions, circulating NP-I levels in healthy adults do not display significant circadian rhythmicity, suggesting stable basal oxytocinergic output. Exploratory findings indicate potential sex-related differences that warrant further investigation.

**Supplementary Information:**

The online version contains supplementary material available at https://doi.org/10.1007/s11102-026-01670-0.

## Introduction

Oxytocin (OXT), a neuropeptide synthesized in the hypothalamic paraventricular and supraoptic nuclei, is well known for its roles in parturition, lactation, social bonding, and stress regulation [1]. Growing evidence highlights its clinical relevance in hypothalamic-pituitary disorders, where OXT deficiency has been identified and associated with heightened anxiety and impaired prosocial behavior [2–6].

Despite increasing clinical interest in OXT, its physiological regulation is poorly understood. Unlike the well-characterized circadian rhythms of neuroendocrine axes such as the hypothalamic-pituitary-adrenal (HPA) and hypothalamic-pituitary-gonadal (HPG) systems, the circadian dynamics of the oxytocinergic system remain unexplored [7–10]. The suprachiasmatic nucleus (SCN) serves as the central circadian pacemaker with arginine vasopressin (AVP) acting as one of the core signals via which the SCN imposes its rhythm on target structures through clock genes and the light/dark cycle regulation [11–13]. Light exposure is the most powerful environmental cue that shifts circadian phase, while the SCN-generated circadian rhythm determines sleep wake timing, with maximum sleep propensity during low body temperature and peak melatonin, and maximum alertness in the evening hours [14]. Emerging evidence suggests that OXT secretion is not static but rather dynamically regulated. In animal models, OXT expression and secretion exhibit diurnal variation, often in synchrony with the light-dark cycle and under the influence of the SCN, the central circadian pacemaker [15–18]. These preclinical studies suggest a complex bidirectional relationship between OXT and other circadian systems, including the melatoninergic axis and sleep regulation, in which melatonin enhances OXT release, and OXT promotes slow-wave sleep [19–21]. In humans, direct evidence for circadian regulation of OXT is limited [22, 23]. Some studies suggest day-night variations in circulating OXT and alterations under circadian disruption, such as sleep deprivation or shift work [24]. However, progress has been hindered by methodological challenges, particularly the poor sensitivity and specificity of OXT assays, which have limited clear interpretation of possible circadian oxytocinergic rhythm [25]. Neurophysin I (NP-I), a carrier protein cleaved from the OXT precursor and co-released in equimolar amounts, offers a more stable and reliable surrogate marker of OXT secretion [26–28]. Understanding the circadian profile of NP-I could thus provide critical insights into OXT system dynamics. This is of particular interest for diagnostic purposes (similar to the requirement for evaluating plasma cortisol in the morning) and for therapeutic approaches (timepoint of OXT replacement to align with circadian dynamics, as with other hormone replacement therapies, e.g., hydrocortisone). However, no study has yet investigated the circadian rhythmicity of NP-I. To address this gap, we conducted a 24-hour observational study in healthy adults with hourly measurements of circulating NP-I, enabling a detailed assessment of the circadian rhythmicity of human oxytocinergic output.

## Methods

### Trial design

This is a secondary analysis of a prospective, observational study conducted at the University Hospital Basel, Switzerland, designed to evaluate the circadian rhythm of neuroendocrine hormones in healthy adults under standardized conditions [29]. The study was approved by the local ethics committee, and written informed consent was obtained from all participants prior to participation.

### Participants

Nineteen healthy volunteers were enrolled. Inclusion criteria required participants to have a normal BMI (18.5–24.9 kg/m²) and be free from chronic somatic or psychiatric diseases. Exclusion criteria included the use of any medications (except for hormonal contraceptives), pregnancy, chronic illnesses, and a history of psychiatric disease. For this secondary analysis, samples from thirteen healthy volunteers were available.

### Study procedure

Participants were instructed to avoid structured physical activity and refrain from sports or vigorous exercise for at least 8 h prior to study inclusion, and to limit fluid intake to ≤ 2000 mL per day for three days preceding the study visit. On the examination day, participants arrived after an overnight fast (minimum 8 h). An intravenous line was placed in the antecubital vein, and blood samples were collected every 60 min for 24 h. During the sampling, starting at 07:30 AM, participants received standardized hospital meals (breakfast [08:05 AM], lunch [12:05 PM], dinner [06:35 PM]) and were allowed to consume up to 1,800 mL of fluids per day, with a maximum of 200 mL per time and 300 mL per hour. Participants remained in the ward throughout the study, under controlled environmental conditions, with natural light during the day and darkened rooms at night. Social and physical activity was restricted, although light movement, such as visiting the toilet or sitting during meals, was permitted. Participants followed their habitual sleep–wake schedule under standardized environmental conditions; no objective (e.g., polysomnography) or subjective sleep assessments were performed.

### Blood samples and biochemical analysis

Samples were collected every hour over a 24-hour period, starting at 07:30 AM and continuing until 07:30 AM the following day, and aliquoted (EDTA plasma, lithium-heparin plasma, and serum). Aliquots were immediately centrifuged at 4 °C at 3000 rpm for 10 min, then stored at -80 °C until batch analysis.

Copeptin was measured using an automated immunofluorescent assay (Kryptor Compact Plus, Thermo Scientific B.R.A.H.M.S.), with a lower detection limit of < 0.7 pmol/L. Cortisol was measured to validate circadian rhythmicity using electrochemiluminescence immunoassay (Elecsys ECLIA). NP-I was quantified by using the Oxytocin-Neurophysin 1 Prepropeptide in vitro SimpleStep sandwich ELISA^®^ immunoassay kit (Abcam, Cambridge, UK) according to the manufacturer’s protocol, with a sensitivity of 10 pg/mL and a detection range of 125–8,000 pg/mL. Unextracted plasma EDTA samples were diluted 1:4 for analysis. The CV for dilution-corrected concentrations demonstrated excellent reproducibility, with an inter-assay CV of 10.0% for plasma samples and an intra-assay CV of 3.2%. Samples exceeding the upper validated assay range (8000 pg/mL) were reanalyzed after appropriate dilution, and the resulting quantitative values were included in the analyses.

### Objectives

The primary aim of the present analysis was to investigate the circadian rhythm of NP-I under standardized conditions in healthy adults. Cortisol served as a reference hormone due to its well-characterized circadian profile.

### Statistical analysis

Statistical analyses were performed using R (version 4.4.3). Continuous variables are reported as median with interquartile range [IQR] or mean with standard deviation (SD), as appropriate.

NP-I concentrations were summarized descriptively at both population and individual levels. Participant-specific 24-hour summary measures (median and mean) were calculated to characterize inter-individual variability. Intra-individual variability was assessed using the within-subject range (maximum–minimum), coefficient of variation (CV), and relative range. Individual peak and nadir times were identified descriptively based on observed maxima and minima, and group-level temporal patterns were summarized using mean concentrations at each timepoint. Circadian variation was modelled with a cosinor nlme: NP‑I_ij_ = (M + b_i_) + (A + a_i_)·cos(ωt_j_ − φ), ω = 2π/24. Random effects on MESOR, amplitude, and phase were systematically evaluated, and models were fitted by maximum likelihood using the nlme package. Model selection was guided by AIC/BIC, with the likelihood ratio test (LRT) applied for nested models. The MESOR, amplitude, and acrophase were estimated with 95% confidence intervals, and rhythmicity was evaluated based on the magnitude and uncertainty of the amplitude. Sex-related differences in NP-I concentrations were explored using subject-specific 24-hour median NP-I values as independent observations. Information on menstrual cycle phase was not collected, and analyses were performed both including and excluding women using oral contraceptives. Copeptin and cortisol concentrations were summarized descriptively at selected timepoints to confirm preserved circadian entrainment. All tests were two-sided, with a significance level of 0.05. As this analysis was exploratory and based on a pre-existing dataset, no formal a priori power calculation for circadian rhythmicity was conducted.

## Results

Between November 1, 2015, and May 1, 2016, thirteen nonsmoking, healthy controls participated in this study. The median age was 23 years [IQR, 19–24] with 46% (*n* = 6) females and a mean BMI of 22.8 kg/m^2^ (SD, 1.6). Two women reported regular use of birth control pills. Baseline characteristics are summarized in Table 1.

**Table 1:** Baseline characteristics

	Overall	By Gender	
	*N* = 13^1^	Male *N* = 7^1^	Female *N* = 6^1^
Age	23.0 (19.0, 24.0)	19.0 (18.0, 24.0)	23.5 (22.0, 26.0)
Ethnicity			
Non-Caucasian	0 (0%)	0 (0%)	0 (0%)
Caucasian	13 (100%)	7 (100%)	6 (100%)
Smoking			
Non-Smoker	13 (100%)	7 (100%)	6 (100%)
Smoker	0 (0%)	0 (0%)	0 (0%)
Hormonal contraceptive	2 (15%)	0 (0%)	2 (33%)
BMI	22.8 (1.6)	23.2 (1.7)	22.3 (1.6)

^1^Median (Q1, Q3); n (%); Mean (SD). Values are presented as median (interquartile range [IQR], Q1–Q3) for non-normally distributed variables, mean (standard deviation [SD]) for normally distributed variables, and n (%) for categorical variables

### Neurophysin-I dynamics

**Fig. 1:**
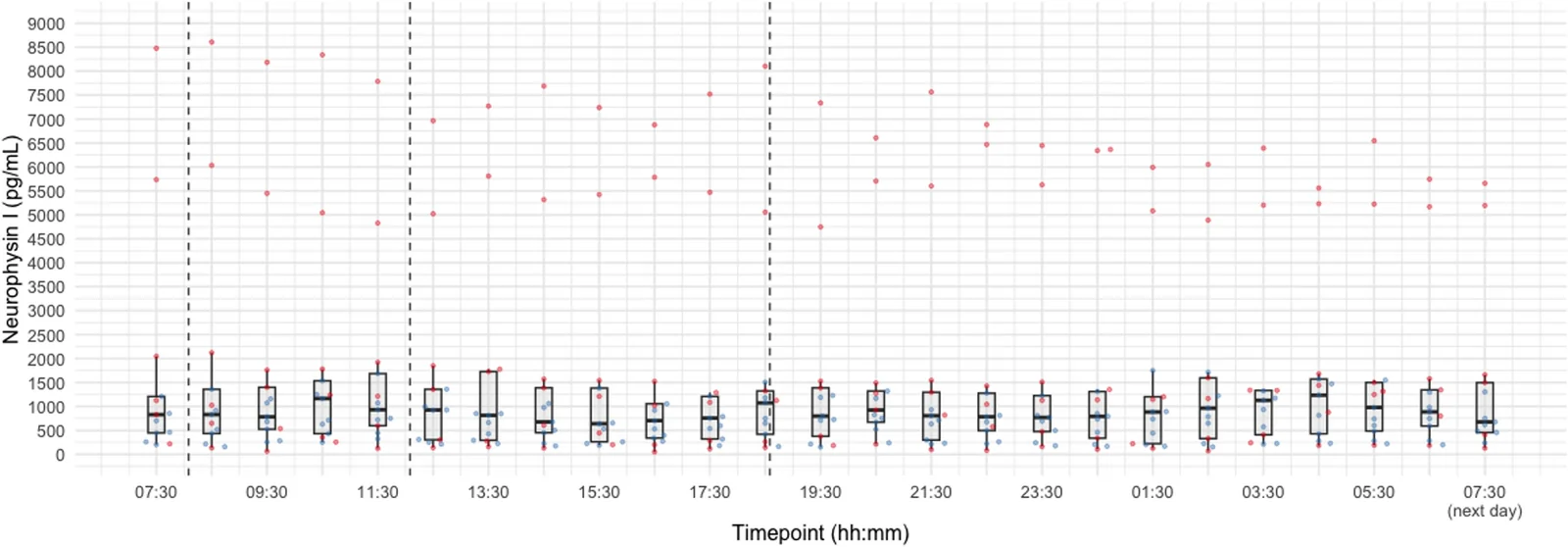
Neurophysin I concentrations over 24 h. The figure shows hourly boxplots of Neurophysin I concentrations (pg/mL) measured from 07:30 AM to 07:30 AM the next day, with every second timepoint (hh: mm) displayed on the x-axis. Individual data points are overlaid using a beeswarm distribution to prevent overlap, with colors representing gender (blue for male, red for female). Dashed vertical lines represent meal times (Breakfast 08:05 AM, Lunch 12:05 PM, Dinner 06:35 PM). The y-axis depicts Neurophysin I concentrations in pg/mL

The plasma NP-I concentrations across all participants are shown in Fig. 1 and summarized in Table 2. Across all individuals, NP-I concentrations ranged from 50 pg/mL to 8,607 pg/mL. The overall median NP-I concentration was 822 pg/mL [339–1,392], and the mean was 1,609 pg/mL (2,084). Inter-individual differences in NP-I concentrations were observed. Participant-specific median 24-hour NP-I concentrations ranged from 143 pg/mL [114–186] to 7,103 pg/mL [6,380–7,714]. The corresponding mean 24-hour NP-I levels ranged from 149 pg/mL (56) to 7,027 pg/mL (997). Intra-individual fluctuations in NP-I concentrations also varied between participants. Across the 24-hour period, participant-specific NP-I ranges (maximum–minimum) ranged from 168 pg/mL to 3,437 pg/mL. Standardized measures of intra-individual NP-I variability demonstrated heterogeneity across participants: the CV ranged from 0.09 to 0.82, and relative ranges (range divided by mean) spanned approximately 0.33 to 3.18.

**Table 2:** Individual Neurophysin I measurements

Subject	Gender	Median	Q1–Q3	Mean	SD	CV (%)	Delta	Max	Time of Max	Min	Time of Min	Hormonal Contraception
S01	Male	814	752–965	868	163	19	555	1,209	17:30	655	15:30	No
S02	Male	1,251	934–1358	1,191	292	25	1,104	1,752	01:30	648	18:30	No
S03	Male	539	485–727	598	182	30	732	983	06:30	251	12:30	No
S04	Female	446	328–648	555	329	59	1,299	1,500	05:30	201	16:30	No
S05	Male	495	432–709	609	331	54	1,413	1,571	04:30	158	08:30	No
S06	Male	848	736–1173	943	281	30	1,068	1,686	11:30	617	07:30 (next day)	No
S07	Male	283	228–455	425	342	81	1,353	1,506	18:30	153	19:30	No
S08	Female	1,524	1353–1760	1,562	246	16	974	2,121	08:30	1,147	01:30	No
S09	Male	213	188–230	215	37	17	168	324	11:30	156	02:30	No
S10	Female	5,470	5083–5744	5,514	493	9	1,798	6,546	05:30	4,748	19:30	Yes
S11	Female	6,965	6341–7689	6,953	1,043	15	3,437	8,607	08:30	5,170	06:30	Yes
S12	Female	1,213	1124–1388	1,263	223	18	909	1,728	13:30	819	21:30	No
S13	Female	140	117–184	148	55	37	206	256	10:30	50	16:30	No

Values are presented as median (Q1–Q3), mean (SD), and coefficient of variation (CV, calculated as [SD/Mean] × 100). Delta refers to the difference between maximum and minimum concentration. Maximum and minimum concentration columns include the respective times of measurement. Hormonal contraception status is indicated as “Yes” or “No”

The timing of the NP-I peaks and minimum concentrations varied across individuals. At the individual level, peak NP-I concentrations occurred most frequently during the early morning. Specifically, five participants reached their peak between 00:00 and 07:30, five between 07:30 and 12:30, two between 12:30 and 18:30, and one between 18:30 and 24:00. The timing of the lowest NP-I concentrations also showed inter-individual variability. Three participants reached their minimum between 00:00 and 07:30; one between 07:30 and 12:30; five between 12:30 and 18:30; and four between 18:30 and 24:00. At the group level, based on mean concentrations across participants at each timepoint, NP-I values reached a maximum at approximately 05:30 and a minimum at approximately 16:30. A mixed-effects cosinor analysis was performed to assess 24-hour circadian variation in circulating NP-I, incorporating subject-specific random intercepts to account for repeated measurements. The model estimated a MESOR (mean level) of 1,604 pg/mL (95% CI [494 to 2,714]). The estimated amplitude of oscillation was small (35 pg/mL) and not statistically significant (95% CI [–110 to 181], *p* > 0.05), indicating the absence of a robust population-level circadian rhythm. The estimated acrophase occurred at 07:01 AM (95% CI [06:08 to 07:53]), suggesting a potential early-morning peak.

Differences in NP-I concentrations were observed between female and male participants. At the individual level, participant-specific median 24-hour NP-I concentrations among females ranged from 143 pg/mL to 7,103 pg/mL, whereas corresponding median values among males ranged from 213 pg/mL to 1,238 pg/mL. Across all timepoints, female participants exhibited higher NP-I concentrations, with a median of 1,372 pg/mL [468–5,344] and a mean of 2,676 pg/mL (2,671), compared with male participants, who had a median concentration of 687 pg/mL [297–932] and a mean of 695 pg/mL (399). Sex-specific rhythm analyses showed no clear or robust circadian pattern differences between females and males (estimated amplitude of oscillation was 120 pg/mL; 95% CI [-163 to 402], in females and − 40 pg/mL; 95% CI [-135 to 55], in males). Notably, two female participants using hormonal contraception exhibited substantially elevated NP-I concentrations, with a mean of 6,234 pg/mL and a median of 6,217 pg/mL, corresponding to approximately four- to five-fold higher levels compared with all other participants.

### Copeptin dynamics and cortisol changes

The overall median copeptin concentration was 2.99 pmol/L [2.43–4.32], and the mean concentration was 3.73 pmol/L (2.18). Intra-individual copeptin fluctuations over the 24-hour period showed moderate variability, with a mean participant-specific range of 4.79 pmol/L (3.55). At the group level, copeptin concentrations peaked in the early morning at approximately 07:30, with a median of 4.40 pmol/L [3.27–6.65]. The lowest group-level copeptin concentrations were observed in the afternoon at approximately 16:30, with a median of 2.74 pmol/L [2.29–3.22]. Cortisol concentrations exhibited the expected diurnal pattern. At midnight, the median cortisol concentration was 43 nmol/L [25–62], increasing by 406 nmol/L [232–443] to 449 nmol/L [294–468] at 07:30.

## Discussion

The main finding of this study is that circulating NP-I does not exhibit relevant circadian rhythmicity over 24 h in healthy adults, supporting that the oxytocinergic system remains stable under unstimulated physiological conditions. Exploratory analyses suggested higher NP-I levels in females, particularly among women using hormonal contraception, highlighting the influence of sex hormones on basal oxytocinergic tone and the need for hormone-sensitive study designs in future research.

The circadian regulation of the oxytocinergic system remains poorly understood, with prior studies yielding mixed findings [15]: Amico et al. observed diurnal rhythmicity in CSF but not in plasma; Forsling et al. identified a nocturnal peak in men; and Baskaran et al. documented pulsatile overnight secretion [15, 30–32]. More recent work remains heterogeneous, ranging from absent rhythmicity in ICU patients to diurnal decreases after awakening during the COVID-19 pandemic [30, 33]. Developmental and psychiatric cohorts have also yielded divergent patterns, including evening elevations in adolescents and increases from awakening to midday in women [24, 34]. Taken together, these studies suggest substantial methodological and population-related heterogeneity, making it difficult to define a consistent circadian pattern. Consistent with our data, at most a small, clinically minor early-morning peak may occur. One possible explanation for these nocturnal fluctuations is modulation by the melatoninergic axis. Melatonin can influence OXT synthesis, release, and receptor expression. Animal studies show predominantly inhibitory effects, whereas in humans, low-dose melatonin may stimulate and high-dose melatonin may inhibit OXT release [19–21, 23]. Against this background, the minor morning peak observed in our data may reflect melatonin–OXT interactions rather than intrinsic circadian control of the system itself. In contrast, cortisol served as a positive control and demonstrated a robust circadian rhythm in all participants. Copeptin levels, used as an indirect marker of AVP activity and SCN-mediated circadian regulation, remained within the expected physiological range. Taken together, these findings support the absence of a pronounced circadian rhythm in NP-I likely reflects characteristics of OXT system dynamics. The prolonged half-life of NP-I may facilitate integration of cumulative OXT secretion and detection of high-amplitude bursts, but may obscure brief, low-amplitude secretory events.

Methodological factors also contribute to inconsistent findings across the literature. OXT is highly labile, prone to rapid degradation, oxidation, and protein binding, making accurate quantification challenging [25, 35]. In contrast, NP-I is stable, easily measurable, and correlates strongly with OXT under stimulated conditions [26, 27]. NP-I rises in physiological oxytocin-mediated states such as parturition and lactation, and evidence demonstrates a strong correlation between stimulated NP-I and OXT following MDMA administration [26, 27, 36, 37]. The present data extend these findings by showing a rather stable intra-individual variability in NP-I under resting conditions.

Our findings also highlight contextual influences, including sex and reproductive hormone status. OXT levels fluctuate across the menstrual cycle, with oestrogen facilitating OXT synthesis, release, and receptor expression at multiple levels [38, 39]. Oestrogen enhances OXT mRNA expression, stimulates dendritic exocytosis in magnocellular neurons, and upregulates OXT receptors in oestrogen-sensitive brain regions [40]. These effects translate into behavioural changes, including social recognition, anxiolysis, and maternal behaviour [41]. An “oestrogen-stimulated neurophysin” has been described in humans, supporting oestrogen-linked modulation of OXT [42–44]. These mechanisms likely contribute to the sex-specific variability observed in our study.

OXT secretion is influenced by pulsatile and state/context-dependent mechanisms. Noradrenergic inputs from somatosensory stimulation (e.g., suckling, parturition), steroid hormones, and kisspeptin all stimulate OXT neurons, whereas GABAergic inhibition, stress, and pain exert complex modulatory effects. Additional contributions arise from histaminergic, dopaminergic, and serotonergic circuits [45–49]. These regulatory pathways support the concept that OXT is released in response to specific physiological and emotional contexts rather than following a robust intrinsic circadian rhythm. The distinction between trait-like and reactive OXT secretion has important diagnostic implications. Basal OXT levels correlate poorly with central release, and basal OXT and NP-I do not differ between healthy individuals and patients with hypothalamic–posterior pituitary dysfunction [5, 50]. Dynamic testing is therefore essential to identify an OXT deficiency. Classical stimulation protocols (exercise, pituitary testing) have shown limited value in eliciting measurable OXT responses [51]. In contrast, MDMA induces potent, reproducible increases in both OXT and NP-I in healthy controls, whereas patients with hypothalamic–posterior pituitary dysfunction exhibit blunted responses [3, 52]. Overall, NP-I shares favourable analytical characteristics with copeptin, the stable AVP precursor that has transformed AVP deficiency diagnostics [53]. Preliminary data suggest that MDMA-stimulated NP-I may even outperform stimulated OXT in differentiating patients from healthy individuals, supporting NP-I as a promising biomarker in future diagnostic algorithms.

From a therapeutic perspective, our findings carry important implications for the development of future replacement strategies. In most replacement therapies, the goal is to replicate the physiological secretion profile as closely as possible. When circadian rhythmicity is present, basal replacement schedules aim to mimic the endogenous rhythm, with supplemental “stress doses” administered during periods of increased demand. OXT treatment, in contrast, has mainly evolved outside such physiological principles. Intranasal OXT is typically given at fixed empirical doses, either as a single bolus before experimental tasks or twice daily in longer-term clinical protocols. By contrast, the absence of circadian rhythmicity in NP-I levels under resting conditions suggests that OXT replacement may not require circadian-based dosing. However, the OXT system shows strong state- and context-dependent reactivity, with release triggered by emotional, social, or stress-related stimuli. This raises the possibility that OXT therapy may benefit from flexible, context-responsive short-acting dosing, complemented by a long-acting formulation to provide a stable physiological background level.

The study has several limitations. First, the relatively small sample size limits statistical power. Although the sampling strategy likely captured physiologically meaningful secretory patterns, oscillations occurring at intervals shorter than one hour cannot be resolved. Second, several factors influencing the oxytocinergic system were not assessed. Individual sleep–wake cycles were not formally recorded, which may have influenced circadian dynamics. Menstrual cycle phase was not documented and hormonal fluctuations across the cycle may affect baseline NP-I concentrations. In addition, metabolic characteristics such as body composition, physical fitness, and eating behavior were not assessed. However, to account for inter-individual baseline variability, we applied mixed-effects cosinor analysis.

In conclusion, although the relatively small sample size limits the ability to exclude subtle circadian variations, our data demonstrate no detectable diurnal rhythm in NP-I levels in healthy adults under basal conditions. These findings support the concept that NP-I—and, by extension, OXT—is predominantly released in response to specific physiological or emotional stimuli rather than governed by a robust intrinsic circadian rhythm. Future studies should systematically examine sex differences, menstrual cycle phase, metabolic characteristics, eating behavior, and context-dependent reactivity.

## Supplementary Information

Supplementary Material 1.

## Data Availability

We may share de-identified, individual participant-level data that underlie the results reported in this Article and related documents, including the study protocol and the statistical analysis plan. Data will be available upon publication of our main manuscript upon receipt of a request detailing the study hypothesis and statistical analysis plan. All requests should be sent to the corresponding author. Based on the scientific rigor of the proposal, the steering committee of this study will discuss all requests and decide whether data sharing is appropriate. All applicants are asked to sign a data access agreement.
