# Supplementary material for: Absence of detectable circadian rhythmicity of neurophysin I, a surrogate marker for oxytocin
Source: Pituitary. 2026 Mar 25;29(2):62. doi: 10.1007/s11102-026-01670-0 (PMC13675142; doi:10.1007/s11102-026-01670-0)
Supplement: Supplementary file 1 [file 11102_2026_1670_MOESM1_ESM.docx]

APPENDIX

**Absence of Detectable Circadian Rhythmicity of Neurophysin I,**

**A Surrogate Marker for Oxytocin**

**Authors**

*Cihan Atila (MD, PhD)^1,2^, *Rakithan Murugesu (MSc)^1,2^, Deborah Rudin (PhD) ^2,3,4^,

Dino Luethi (PhD) ^2,3,4^, Matthias E Liechti (MD)^2,3,4^, and Mirjam Christ-Crain (MD, PhD)^1,2^

**Affiliations**

^1^Department of Endocrinology, Diabetology and Metabolism, University Hospital Basel, Basel, Switzerland

^2^Department of Clinical Research, University of Basel, University Hospital Basel, Basel, Switzerland

^3^Division of Clinical Pharmacology and Toxicology, University Hospital Basel, Basel, Switzerland

^4^Department of Biomedicine, University of Basel, University Hospital Basel, Basel, Switzerland

*These authors contribute equally and share first authorship

**Short title**: Circadian Rhythm of Neurophysin I

**Keywords:** biomarker, hypothalamic regulation, posterior pituitary, suprachiasmatic nucleus, vasopressin, sleep-wake regulation, melatonin, social behaviour

**Correspondence**

Dr. Cihan Atila

Department of Endocrinology, Diabetes and Metabolism, University Hospital Basel

Petersgraben 4, 4031 Basel, Switzerland

Phone: +41 61 265 50 78, e-mail: cihan.atila@usb.ch

**Word count** (Introduction, methods, results, and discussion): 2600

**Table 3 Median Neurophysin I concentrations (pg/mL) and related metrics across different groups**

| Group | N | Median | Q1 – Q3 | Delta (Median) | Max Conc. | Time of Max | Min Conc. | Time of Min |
| --- | --- | --- | --- | --- | --- | --- | --- | --- |
| Overall | 13 | 814 | 446 – 1251 | 6,825 | 2,291 | 05:30 | 1,136 | 16:30 |
| Male | 7 | 539 | 389 – 831 | 1,038 | 1,290 | 11:30 | 377 | 02:30 |
| Female | 6 | 1,368 | 638 – 4483 | 6,825 | 3,460 | 05:30 | 2,023 | 16:30 |
| No hormonal contraceptive | 11 | 539 | 364 – 1031 | 1,384 | 1,331 | 11:30 | 441 | 16:30 |
| Hormonal contraceptive | 2 | 6,217 | 5844 – 6591 | 1,495 | 7,577 | 05:30 | 4,959 | 06:30 |
| Female \| No Hormonal Contraceptive | 4 | 829 | 369 – 1291 | 1,384 | 1,401 | 05:30 | 554 | 16:30 |

Table 3: The table presents the Neurophysin I concentrations (pg/mL) for the overall population (n = 13) and subgroups stratified by sex and hormonal contraceptive use. Values are reported as median (Q1–Q3). "Delta (Median)" refers to the difference between the median maximum and median minimum Neurophysin I concentrations. The table also includes the maximum and minimum Neurophysin I concentrations and their respective times of occurrence. Groups include "Male," "Female," "No hormonal contraceptive," "Hormonal contraceptive" (two of six females), and "Female and No hormonal contraceptive."

**Table 4 Mean Neurophysin I concentrations (pg/mL) and related metrics across different groups**

| Group | N | Mean | SD | CV (%) | Delta (Mean) | Max Conc. | Time of Max | Min Conc. | Time of Min |
| --- | --- | --- | --- | --- | --- | --- | --- | --- | --- |
| Overall | 13 | 1,603 | 2,116 | 132 | 6,805 | 2,291 | 05:30 | 1,136 | 16:30 |
| Male | 7 | 693 | 331 | 48 | 976 | 1,290 | 11:30 | 377 | 02:30 |
| Female | 6 | 2,666 | 2,845 | 107 | 6,805 | 3,460 | 05:30 | 2,023 | 16:30 |
| No hormonal contraceptive | 11 | 762 | 447 | 59 | 1,413 | 1,331 | 11:30 | 441 | 16:30 |
| Hormonal contraceptive | 2 | 6,234 | 1,018 | 16 | 1,439 | 7,577 | 05:30 | 4,959 | 06:30 |
| Female \| No Hormonal Contraceptive | 4 | 882 | 646 | 73 | 1,413 | 1,401 | 05:30 | 554 | 16:30 |

Table 4: The table shows the Neurophysin I concentrations (pg/mL) for the overall population (n = 13) and subgroups stratified by sex and hormonal contraceptive use. Values are reported as mean ± standard deviation (SD). The coefficient of variation (CV) is calculated as [(SD/Mean) × 100]. "Delta (Mean)" refers to the difference between the mean maximum and mean minimum Neurophysin I concentrations. The table also includes the maximum and minimum Neurophysin I concentrations and their respective times of occurrence. Groups include "Male," "Female," "No hormonal contraceptive," "Hormonal contraceptive" (two of six females), and "Female and No hormonal contraceptive".

**Table 5 Rhythmic parameters from the non-linear mixed effects cosinor model analysis of Neurophysin I**

| Parameter | Estimate | 95% CI |
| --- | --- | --- |
| Mesor | 1604 pg/mL | 494 - 2714 pg/mL |
| Amplitude | 35 pg/mL | -110 - 181 pg/mL |
| Phase (clock time) | 07:01 | 06:08 - 07:53 |

Table 5 The table presents the rhythmic parameters derived from the non-linear mixed effects cosinor model for Neurophysin I concentrations (pg/mL). Parameters include the mesor (mean level around which the rhythm oscillates), amplitude (half the peak-to-trough variation), and phase (timing of the peak in clock time). Values are reported as estimates with their corresponding 95% confidence intervals (CI).

**Table 6 Rhythmic parameters from the non-linear mixed effects cosinor model analysis of Neurophysin I across subgroups**

| Group | Parameter | Estimate | 95% CI |
| --- | --- | --- | --- |
| Overall | Mesor | 1604 pg/ml | 494 - 2714 pg/ml |
|  | Amplitude | 35 pg/ml | -110 - 181 pg/ml |
|  | Phase (clock time) | 07:00 | 06:07 - 07:53 |
| Male | Mesor | 692 pg/ml | 464 - 920 pg/ml |
|  | Amplitude | -40 pg/ml | -135 - 55 pg/ml |
|  | Phase (clock time) | 08:14 | 06:17 - 10:11 |
| Female | Mesor | 2667 pg/ml | 570 - 4764 pg/ml |
|  | Amplitude | 120 pg/ml | -163 - 402 pg/ml |
|  | Phase (clock time) | 06:53 | 05:42 - 08:03 |
| No Hormonal Contraceptive | Mesor | 761 pg/ml | 508 - 1014 pg/ml |
|  | Amplitude | -38 pg/ml | -104 - 28 pg/ml |
|  | Phase (clock time) | 08:20 | 06:27 - 10:14 |
| Hormonal Contraceptive | Mesor | 6237 pg/ml | 5206 - 7268 pg/ml |
|  | Amplitude | 348 pg/ml | -389 - 1086 pg/ml |
|  | Phase (clock time) | 06:59 | 05:06 - 08:53 |
| Female No Hormonal Contraceptive | Mesor | 878 pg/ml | 324 - 1433 pg/ml |
|  | Amplitude | 98 pg/ml | 27 - 169 pg/ml |
|  | Phase (clock time) | 01:36 | 23:10 - 04:02 |

Table 6: The table presents the rhythmic parameters derived from the non-linear mixed effects cosinor model for Neurophysin I concentrations (pg/mL) across different subgroups stratified by gender and hormonal contraceptive use. Parameters include the mesor (mean level around which the rhythm oscillates), amplitude (half the peak-to-trough variation), and acrophase (timing of the peak in clock time, 24-hour format). Values are reported as estimates with their corresponding 95% confidence intervals (CI). Subgroups analyzed include the "Overall" sample, "Male," "Female," "No hormonal contraceptive," "Hormonal contraceptive" (two of six females), and "Female and No hormonal contraceptive".

**Table 7 Model comparison based on AIC, BIC, Log-Likelihood and degrees of freedom**

| Modell | AIC | BIC | LogLik | df |
| --- | --- | --- | --- | --- |
| **Non-linear mixed effects with random effect on Mesor + Amplitude** | **4,852** | **4,879** | **-2,419** | **7** |
| Non-linear mixed effects with random effect on Mesor + Amplitude + Phase | 4,858 | 4,896 | -2,419 | 10 |
| Linear Mixed-Effect | 4,864 | 4,883 | -2,427 | 5 |
| Non-linear mixed effects with random effect on Mesor only | 4,896 | 4,915 | -2,443 | 5 |
| Non-linear mixed effects with random effect on Mesor + Phase | 4,900 | 4,927 | -2,443 | 7 |
| Non-linear mixed effects with random effect on Phase only | 5,895 | 5,914 | -2,942 | 5 |
| Non-linear mixed effects with random effect on Amplitude only | 5,895 | 5,914 | -2,942 | 5 |
| Non-linear mixed effects with random effect on Amplitude + Phase | 5,899 | 5,925 | -2,942 | 7 |
| *AIC = Akaike Information Criterion BIC = Bayesian Information Criterion LogLik = Log-Likelihood df = degrees of freedom* | | | | |

Table 7: This table compares different statistical models fitted to the circadian rhythm data of Neurophysin I. The models are evaluated using Akaike Information Criterion (AIC), Bayesian Information Criterion (BIC), Log-Likelihood (LogLik), and degrees of freedom (df). Models with lower AIC and BIC values indicate better goodness-of-fit. Fixed and random effects terms, such as Mesor (M), Amplitude (A), and Phase (φ), are incorporated in specific models, including a linear mixed-effect model (LME) for reference.

**Table 8 Copeptin (pmol/L) measurements and related variables for individual study participants**

| Subject | Gender | Median (pmol/L) | Q1–Q3 (pmol/L) | Mean (pmol/L) | SD (pmol/L) | CV (%) | Delta (pmol/L) | Max (pmol/L) | Time of Max | Min (pmol/L) | Time of Min | Hormonal Contraception |
| --- | --- | --- | --- | --- | --- | --- | --- | --- | --- | --- | --- | --- |
| S01 | Male | 3.23 | 2.88 – 4.49 | 3.66 | 1.27 | 34.83 | 4.99 | 6.98 | 20:30 | 1.99 | 05:30 | No |
| S02 | Male | 6.04 | 5.35 – 7.08 | 6.15 | 1.62 | 26.42 | 7.17 | 10.09 | 07:30 | 2.92 | 19:30 | No |
| S03 | Male | 3.43 | 2.86 – 4.07 | 3.69 | 1.13 | 30.54 | 4.42 | 6.65 | 07:30 | 2.23 | 01:30 | No |
| S04 | Female | 3.11 | 2.5 – 3.35 | 3.03 | 0.57 | 18.88 | 2.22 | 4.28 | 03:30 | 2.06 | 10:30 | No |
| S05 | Male | 3.03 | 2.7 – 3.41 | 3.11 | 0.66 | 21.12 | 2.60 | 4.58 | 10:30 | 1.97 | 08:30 | No |
| S06 | Male | 3.71 | 3.15 – 5.14 | 4.17 | 1.23 | 29.55 | 4.17 | 6.45 | 04:30 | 2.28 | 11:30 | No |
| S07 | Male | 4.51 | 3.56 – 6.07 | 4.99 | 1.50 | 30.10 | 4.75 | 7.69 | 06:30 | 2.94 | 20:30 | No |
| S08 | Female | 2.57 | 2.16 – 2.9 | 2.55 | 0.47 | 18.48 | 1.69 | 3.55 | 08:30 | 1.85 | 16:30 | No |
| S09 | Male | 2.11 | 1.81 – 2.45 | 2.20 | 0.56 | 25.40 | 2.46 | 3.92 | 07:30 | 1.45 | 15:30 | No |
| S10 | Female | 2.19 | 2.08 – 2.44 | 2.26 | 0.39 | 17.23 | 1.54 | 3.10 | 15:30 | 1.56 | 19:30 | Yes |
| S11 | Female | 2.47 | 2.14 – 2.71 | 2.51 | 0.49 | 19.45 | 2.35 | 4.31 | 13:30 | 1.96 | 10:30 | Yes |
| S12 | Female | 3.47 | 2.71 – 5.09 | 3.93 | 1.45 | 36.79 | 4.20 | 6.40 | 07:30 | 2.20 | 22:30 | No |
| S13 | Female | 15.27 | 2.55 – 47.85 | 30.31 | 30.09 | 99.29 | 86.26 | 88.00 | 07:30 (next day) | 1.74 | 16:30 | No |
| Mean = average concentration; SD = standard deviation; CV = coefficient of variation [CV = (SD / Mean) * 100]; Delta = max – min; Q1–Q3 = interquartile range; Time of Max/Min = time at which the max/min occurred | | | | | | | | | | | | |

Table 8: The table presents the measured Copeptin concentrations (pmol/L) for each individual. Values are reported as median (Q1–Q3), mean (SD), and coefficient of variation (CV, calculated as [(SD/Mean) × 100]). "Delta" refers to the difference between the maximum and minimum Copeptin concentrations. The table also includes the maximum and minimum Copeptin concentrations and their respective times of occurrence. Hormonal contraception status is indicated as "Yes" or "No."

**Table 9 Median Copeptin concentrations (pmol/L) and related metrics across different groups**

| Group | N | Median | Q1 – Q3 | Delta (Median) | Max Conc. | Time of Max | Min Conc. | Time of Min |
| --- | --- | --- | --- | --- | --- | --- | --- | --- |
| Overall | 13 | 3.23 | 2.57 – 3.71 | 13.15 | 12.00 | 7:30 AM | 2.09 | 10:30 AM |
| Male | 7 | 3.43 | 3.13 – 4.11 | 3.92 | 6.62 | 7:30 AM | 2.26 | 1:30 AM |
| Female | 6 | 2.84 | 2.5 – 3.38 | 13.08 | 18.27 | 1:30 PM | 1.90 | 10:30 AM |
| No hormonal contraceptive | 11 | 3.43 | 3.07 – 4.11 | 13.15 | 13.51 | 7:30 AM | 2.15 | 4:30 PM |
| Hormonal contraceptive | 2 | 2.33 | 2.26 – 2.4 | 0.28 | 3.70 | 1:30 PM | 1.76 | 10:30 AM |
| Female \| No Hormonal Contraceptive | 4 | 3.29 | 2.98 – 6.42 | 12.69 | 25.56 | 3:30 AM | 1.97 | 4:30 PM |

Table 9: The table presents the Copeptin concentrations (pmol/L) for the overall population (n = 13) and subgroups stratified by sex and hormonal contraceptive use. Values are reported as median (Q1–Q3). "Delta (Median)" refers to the difference between the median maximum and median minimum Copeptin concentrations. The table also includes the maximum and minimum Copeptin concentrations and their respective times of occurrence. Groups include "Male," "Female," "No hormonal contraceptive," "Hormonal contraceptive" (two of six females), and "Female and No hormonal contraceptive."

**Table 10 Mean Copeptin concentrations (pmol/L) and related metrics across different groups**

| Group | N | Mean | SD | CV (%) | Delta (Mean) | Max Conc. | Time of Max | Min Conc. | Time of Min |
| --- | --- | --- | --- | --- | --- | --- | --- | --- | --- |
| Overall | 13 | 5.58 | 7.52 | 134.67 | 28.11 | 12.00 | 7:30 AM | 2.09 | 10:30 AM |
| Male | 7 | 4.00 | 1.28 | 32.12 | 3.95 | 6.62 | 7:30 AM | 2.26 | 1:30 AM |
| Female | 6 | 7.43 | 11.22 | 151.03 | 28.05 | 18.27 | 1:30 PM | 1.90 | 10:30 AM |
| No hormonal contraceptive | 11 | 6.16 | 8.09 | 131.20 | 28.11 | 13.51 | 7:30 AM | 2.15 | 4:30 PM |
| Hormonal contraceptive | 2 | 2.38 | 0.18 | 7.36 | 0.25 | 3.70 | 1:30 PM | 1.76 | 10:30 AM |
| Female \| No Hormonal Contraceptive | 4 | 9.96 | 13.58 | 136.43 | 27.76 | 25.56 | 3:30 AM | 1.97 | 4:30 PM |

Table 10: The table shows the Copeptin concentrations (pmol/L) for the overall population (n = 13) and subgroups stratified by sex and hormonal contraceptive use. Values are reported as mean ± standard deviation (SD). The coefficient of variation (CV) is calculated as [(SD/Mean) × 100]. "Delta (Mean)" refers to the difference between the mean maximum and mean minimum Copeptin concentrations. The table also includes the maximum and minimum Copeptin concentrations and their respective times of occurrence. Groups include "Male," "Female," "No hormonal contraceptive," "Hormonal contraceptive" (two of six females), and "Female and No hormonal contraceptive."

**Table 11 Rhythmic parameters from the non-linear mixed effects cosinor model analysis of Copeptin**

| Parameter | Estimate | X95..CI |
| --- | --- | --- |
| Mesor | 5.6 pmol/L | 1.5 - 9.7 pmol/L |
| Amplitude | 1.8 pmol/L | 0.7 - 3.2 pmol/L |
| Phase (clock time) | 05:23 | 02:25 - 08:36 |

Table 11: The table presents the rhythmic parameters derived from the non-linear mixed effects cosinor model for Copeptin concentrations (pmol/L). Parameters include the mesor (mean level around which the rhythm oscillates), amplitude (half the peak-to-trough variation), and phase (timing of the peak in clock time). Values are reported as estimates with their corresponding 95% confidence intervals (CI).

**Table 12 Median concentrations at midnight and in the morning across all subjects**

| Timepoint | Subjects (n) | Median (nmol/L) | Q1 - Q3 |
| --- | --- | --- | --- |
| Midnight (24:00) | 13 | 43 | 25 - 62 |
| Morning (07:30) | 13 | 449 | 294 - 468 |

Table 12: The table presents the median concentrations (nmol/L) of all 13 subjects at two timepoints: midnight (24:00) and the following morning (07:30). Values are reported as median (Q1–Q3). The number of subjects (n = 13) is consistent across both timepoints.

**Figure 2 Non-linear mixed effects cosinor analysis of Neurophysin I concentrations (pg/mL) over 24 hours**

Figure 2: The figure shows the calculated population mean (black line) for Neurophysin I concentrations (pg/mL) based on the non-linear mixed effects cosinor analysis. The measured Neurophysin I values for each subject are plotted at the respective timepoints, with each subject represented by a unique color and point shape. Timepoints (hh:mm) are displayed every two hours on the x-axis. Dashed vertical lines mark meal times, which are also included in the legend, along with the population mean and subject identifiers.

| **Figure 3 Copeptin concentrations (pmol/L) over 24 hours** | **Figure 4 Cortisol concentrations (nmol/L) measured at midnight and the following morning** |
| --- | --- |
|   Figure 3: The figure shows boxplots of Copeptin concentrations (pmol/L) measured hourly from 07:30 AM to 07:30 AM the next day. Timepoints (hh:mm) are displayed every two hours.. Dashed vertical lines represent meal times at their respective hours (Breakfast 08:05 AM, Lunch 12:05 PM, Dinner 6:35 PM).. The y-axis shows Copeptin concentrations in pmol/L. |   Figure 4: The figure shows boxplots of cortisol concentrations (nmol/L) measured at two timepoints: midnight (24:00) and 07:30 AM the following day. The y-axis displays cortisol concentrations in nmol/L. |
